# Neutrophil‐reactive autoantibodies in blood donors: Can we expect TRALI?

**DOI:** 10.1111/trf.70125

**Published:** 2026-02-06

**Authors:** Stefanie Jehle, Darvin Preuss, Yannick Waxmann, Anja Spies‐Naumann, Silke Schmidt, Rick Kapur, Ulrich J. Sachs, Behnaz Bayat

**Affiliations:** ^1^ Institute for Clinical Immunology, Transfusion Medicine, and Haemostaseology Justus‐Liebig‐University Giessen Germany; ^2^ Sanquin Blood Supply Foundation Department Research, Amsterdam UMC, Location University of Amsterdam, Landsteiner Laboratory Amsterdam the Netherlands; ^3^ Center for Transfusion Medicine and Haemotherapy, Incorporating the Department of Thrombosis and Haemostasis and the European Haemophilia Comprehensive Care Center Giessen and Marburg University Hospital Giessen Germany

## Abstract

**Background:**

Transfusion‐related acute lung injury (TRALI) is a clinical condition characterized by acute non‐cardiogenic pulmonary edema during or after transfusion. Despite several mitigation strategies, TRALI remains a leading cause of transfusion‐related deaths. Tests on blood donors involved in TRALI, apart from leukocyte/endothelial reactive alloantibodies, can also reveal the presence of neutrophil autoantibodies. Here, we ask the question whether these autoantibodies could play a role in the development of TRALI.

**Materials and methods:**

Sera (*n* = 15) from donors involved in TRALI and not containing alloantibodies against human neutrophil antigens (HNAs) and human leukocyte antigens (HLAs) were collected if their serological characterization was suggestive for the presence of neutrophil‐reactive autoantibodies. Sera (*n* = 12) were suitable for the study. Their ability to bind complement, to activate neutrophils, and to disrupt the endothelial barrier using albumin influx through an endothelial monolayer in a transwell chamber in the presence and absence of neutrophils was investigated.

**Results:**

None of the AIN sera, but 2/12 TRALI sera, induced reactive oxygen species (ROS) in neutrophils. Both of these TRALI sera induced endothelial barrier permeability in the presence, but not in the absence, of neutrophils. These two sera did not activate complement.

**Conclusion:**

Autoantibodies against neutrophils present in transfused blood components appear to be capable of contributing to TRALI in selected but not in all investigated cases, based on their ability to activate neutrophil ROS and induce endothelial cell permeability in vitro. Further analysis is required to understand the potential functional effects of neutrophil autoantibodies in TRALI.

## 1 INTRODUCTION

Transfusion‐related acute lung injury (TRALI) is a serious clinical condition characterized by acute non‐cardiogenic pulmonary edema that occurs during or after transfusion.^1^, ^2^ The pathophysiology has proven to be complex.^3^ Based on etiology, two main types of TRALI can be distinguished: antibody‐independent and antibody‐dependent TRALI. The main cause of the first type is bio‐reactive molecules generated during storage which induce endothelial or neutrophil activation and lead to endothelial barrier break‐down and pulmonary edema. Responsible for the second type are alloantibodies against human neutrophil antigens (HNAs) or human leukocyte antigens (HLA).^4^, ^5^ Binding of alloantibodies to target antigens expressed on recipients' neutrophils or endothelial cells induces cellular activation which can also lead to endothelial barrier break‐down.

TRALI induction in recipients follows a two‐hit model, in which patient predisposition (the first hit) and transfusion (the second hit) combine to reach a threshold capable of inducing TRALI.^6^ In antibody‐dependent TRALI, binding of transfused antibodies (the second hit) to cognate antigens in pre‐activated cells (the first hit) induces massive activation of target cells. These target cells then interact with pre‐activated endothelial cells (the first hit), leading to endothelial barrier dysfunction and, consequently, lung edema, which is implicated in TRALI.

HNA or HLA incompatibility between mother and child during pregnancy or between recipient and donor in the event of a transfusion or transplantation can induce alloimmunization.^7^ Alloantibodies present in the bloodstream of a blood donor are collected in blood products during blood donation. The highest amount of antibodies is present in plasma, and plasma was the blood component most frequently involved in TRALI.^8^ Later, a male‐only plasma strategy was introduced in most Western countries where only plasma obtained from male donors without a history of transfusion was used clinically.^9^, ^10^ After adopting this strategy, the number of TRALI cases has dropped significantly.^11^ Despite this successful intervention, TRALI remains a leading cause of transfusion‐related deaths.^12^ The question must be raised as to which other, previously overlooked influencing factors might cause TRALI.

In the past, when investigating suspected TRALI cases serologically, we gained the impression that the retained samples of blood donors occasionally appeared to contain antibodies against neutrophils which serologically behave like autoantibodies. It is unknown whether such autoantibodies can trigger an immunological reaction in the recipient's body. Therefore, in the prospective 2‐year study presented here, all suspected TRALI cases in which the donor material appeared to contain neutrophil autoantibodies were investigated in detail in order to provisionally approach the question of whether such antibodies may have the ability to trigger TRALI.

## 2 MATERIALS AND METHODS

### 2.1 Patient material

As part of the standard procedure, serum samples involved in TRALI, as well as samples suspected of being associated with antibody‐mediated neutropenia, were analyzed employing standard assays (granulocyte immunofluorescence [GIFT], granulocyte agglutination [GAT], and lymphocyte immunofluorescence [LIFT] test) using HNA‐typed neutrophils from four donors. Positive reactivity in GIFT is considered to indicate the presence of neutrophil‐reactive antibodies.^13^ Samples showing positive reactivity in GIFT underwent further analysis using a monoclonal antibody immobilization of granulocyte antigens (MAIGA) test to determine the specificities of the neutrophil‐reactive antibodies.^14^ Based on the patient's HNA genotype, specific reactivity against a particular HNA allele in MAIGA was considered to be an alloantibody. Samples showing no specific reactivity against a particular HNA allele were identified as autoantibodies. It should be noted, however, that autoantibodies of anti‐HNA‐1a “pseudspecificty” are sometimes implicated in primary autoimmune neutropenia in children.^15^ To investigate the presence or absence of HLA antibodies, all samples were examined using LabScreen (LABScreen PRA Class I and class II, Thermo Fisher Scientific, Darmstadt, Germany) on a Luminex system.

For the current study, the leftover material from donors (without HLA or HNA alloantibodies) involved in suspected cases of TRALI (*n* = 15) and leftover material from patients diagnosed with autoimmune neutropenia (*n* = 9) was used. Among the TRALI samples, 3 samples contained antibodies against HLA and HNA, and 12 samples contained HNA autoantibodies only. Autoimmune sera were selected accordingly. Sera from blood donors with previously identified alloantibodies and sera from blood donors without detectable antibodies against HLA and neutrophils were used as positive and negative controls, respectively.

### 2.2 Antibodies and reagents

Monoclonal antibodies (mAbs) and reagents used in this study are listed with their source in brackets as follows: Hybridoma clone 7D8, producing a mAb against CD177, was a kind gift from Dr. David Stroncek (National Institutes of Health, Bethesda, MD, USA). Hybridoma cells were cultured in RPMI with 10% fetal calf serum (FCS) and 1% penicillin/streptomycin (P/S) (Invitrogen, Karlsruhe, Germany). Anti‐CD177 (clone MEM‐166, Serotec, Duesseldorf, Germany), anti‐CD11a (clone B‐B15, Medix Biochemica, Konstanz, Germany), anti‐CD11b (clone Mac‐1, Beckman Coulter, Krefeld, Germany), anti‐CD18 (clone ITGB2, Biozol Diagnostica, Hamburg, Germany), anti‐CD16b (clone LNK16, Santa Cruz Biotechnology, Heidelberg, Germany; clone 3G8, Beckman Coulter, Krefeld, Germany) and anti‐HLA class I (clone W6/32, Biolegend, Koblenz, Germany); *ortho*‐pheneylendiamine (Dako, Hamburg, Germany); lymphocyte separation media (Anprotec, Bruckberg, Germany); CM‐H_2_DCFDA (Thermofisher, Darmstadt, Germany); human C1q complement protein (Merck, Darmstadt, Germany); polyclonal sheep anti‐human C1q FITC mAb (Bio‐Rad, Dreieich, Germany); fluorescein‐isothiocyanate‐labeled bovine serum albumin (FITC‐BSA, Taufkirchen, Germany); fibronectin (Merck, Darmstadt, Germany); FITC‐labeled rabbit anti‐human IgG antibody (Jackson ImmunoResources, Brussels, Belgium); Phorbol 12‐myristate 13‐acetate (Merck, Darmstadt, Germany); paraformaldehyde (PFA) (Carl Roth, Karlsruhe Germany); Dulbecco's Modified Eagle Medium (DMEM) (Anprotec, Bruckberg, Germany); and EBM‐2 bullet kit (Lonza, Cologne, Germany).

### 2.3 Neutrophil serology

Antibodies were detected in sera with a combination of GAT, GIFT test, and lymphocyte immunofluorescence test (LIFT) as described previously.^9^ In brief, neutrophils and lymphocytes of various donors typed for the known neutrophil antigens and HLA Class I were isolated with dextran and subsequent density gradient centrifugation. For GAT, neutrophils were incubated with serum for 2 h at room temperature, and agglutination was evaluated by microscopy. For the immunofluorescence tests, neutrophils and lymphocytes were fixed with PFA. Cells were incubated with serum, washed, and stained with fluorescein isothiocyanate (FITC)‐conjugated rabbit anti‐human IgG. Cell‐bound fluorescence was assessed either by fluorescence microscopy (GIFT) or flow cytometry (LIFT). Sera containing previously identified HNA antibodies and sera from normal healthy donors served as positive and negative controls, respectively.

### 2.4 mAb immobilization of granulocyte antigens assay (MAIGA)

MAIGA was performed as previously described.^10^ Briefly, fixed neutrophils from HNA‐typed donors were incubated with human serum (undiluted, 1:10 dilution) for 30 min at 37°C. After being washed, cells were incubated with specific mAbs (anti‐CD177, anti‐CD16b, anti‐CD11b, anti‐CD11a, anti‐CD18, anti‐HLA I) for 30 min at 37°C. Cells were washed and solubilized with lysis buffer (1% Triton X‐100, 5 mmol/L ethylenediaminetetraacetate (EDTA), 2 mmol/L phenylmethylsulfonyl fluoride, 150 mmol/L NaCl in 20 mmol/L Tris buffer, pH 7.4) for 30 min at 4°C. After sonification and centrifugation at 15,000 g for 30 min, 70‐μL supernatant was diluted with 180‐μL wash buffer. Aliquots of diluted supernatant were transferred to a microtiter well coated with goat anti‐mouse immunoglobulin IgG. Goat anti‐human IgG conjugated with peroxidase was added. The reaction was then visualized by adding a substrate system containing ortho‐phenylenediamine (Dako, Hamburg, Germany), stopped, and measured at 492 nm in an enzyme‐linked immunosorbent assay (ELISA) reader (Tecan, Crailsheim, Germany). Nine samples contain only HNA autoantibodies. Autoimmune sera were selected according to serological results obtained with TRALI samples. Sera from blood donors with previously identified alloantibodies and sera from blood donors without detectable antibodies against HLA and neutrophils were used as positive and negative controls, respectively.

### 2.5 Detection of HLA class I antibodies

To exclude the presence of HLA antibodies, sera were further analyzed in a commercially available HLA class I and class II screening assay. After incubation of serum with beads (LABScreen PRA Class I and class II, Thermo Fisher Scientific, Darmstadt, Germany), fluorescent emission from beads was analyzed on a Luminex system (Luminex Corporation, Austin, TX, USA). Sera containing anti‐HLA antibodies were excluded from the study.

### 2.6 Cell culture

Ea.hy 926 cell line was purchased from ATCC (Manassas, VA, USA) and cultured in DMEM with 10% FCS and 1% penicillin–streptomycin (P/S) at 37°C, 5% CO_2_. Pooled human umbilical vein endothelial cells (HUVECs) were purchased from Lonza (Cologne, Germany) and cultured from P1 to P5 in EBM‐2 medium with 10% FCS and 1% P/S.

### 2.7 IgG binding to Ea.hy 926 cells

To evaluate the binding capacity of human IgGs present in TRALI sera, 3 × 10^5^ Ea.hy 926 cells were incubated with 25‐μL serum. After washing, bound human IgGs were detected using a FITC‐labeled secondary anti‐human IgG antibody (rabbit anti‐human‐IgG) (1:500 dilution) and analyzed in flow cytometry. A serum containing alloantibodies reactive with endothelial cells and serum from a healthy donor were used as positive and negative controls, respectively.

### 2.8 Production of reactive oxygen species (ROS)

To evaluate the capability of sera to induce ROS, 1 × 10^6^ isolated neutrophils in resting or fMLP (10^−7^ M, 43.75 ng/mL for 30 min) stimulated conditions or 3 × 10^5^ Ea.hy 926 were incubated with 20 μL serum for 1 h at room temperature (RT). ROS production was determined using 1 μM 2′,7′‐dichlorodihydrofluorescein‐diacetate (H_2_DCFDA) by flow cytometry. Phorbol 12‐myristate 13‐acetate (PMA, 10 nM) were used as positive control.

### 2.9 Complement fixation assay

Sera were tested for complement activation via C1q binding to neutrophils. Resting or fMLP (10^−7^ M, 43.75 ng/mL, for 30 min) preincubated cells were fixed with 1% PFA and incubated with serum (1:3 diluted) for 30 min at RT. After washing, 2.5‐μg human C1q protein was added for 20 min at 37°C. Bound C1q was detected by FITC‐labeled polyclonal anti‐human C1q (1:100 dilution) on a flow cytometer. Heat‐inactivated AB serum was used as negative control.

### 2.10 Endothelial permeability assay

The effect of serum in the presence and absence of neutrophils on endothelial barrier integrity was evaluated in a permeability assay using 24‐transwell chambers (pore size 0.4 μm; Greiner Bio‐one, Frickenhausen, Germany). The upper chamber was coated with 50 ng/mL fibronectin for 1 h at 37°C. For each experiment, 5 × 10^4^ HUVEC were seeded in the upper chamber and allowed to form a confluent monolayer for 24 h at 37°C, 5% CO_2_. In some wells, endothelial cells were stimulated with 20 ng/mL TNF‐α for 18 h. Formation of the monolayer was confirmed by light microscopy. Neutrophils (2 × 10^5^) were incubated with 20‐μL serum and transferred to the upper chamber in the presence of 5 mg/mL FITC‐BSA. After 180 min, the concentration of FITC‐BSA in the lower chamber was measured using a microplate fluorescence reader (FLX 800, Bio‐Tek instruments).

### 2.11 Statistics

Statistical analysis was performed using Prism 8 (GraphPad Software, Inc., La Jolla, CA, USA). A *p* < 0.05 was considered to indicate statistical significance.

## 3 RESULTS

### 3.1 Analysis of sera from donors involved in TRALI

Serum samples from donors involved in TRALI are received in our laboratory for detection of HNA and HLA alloantibodies. Over a 2‐year period, sera from 96 donors involved in 27 TRALI cases were investigated. Among TRALI cases, 12 samples did not show positive reactivity in GIFT and were therefore excluded from the cohort. In 15 TRALI cases with positive GIFT results, none of the involved donor sera contained alloantibodies against HNA or HLA. Out of these, three samples contained antibodies against HLA/HNA, and 12 samples contained autoantibodies against neutrophils. Ten of the 12 sera (containing alloantibodies) also showed positive MAIGA reactivity. The detection of neutrophil‐reactive antibodies by GIFT or by GIFT and MAIGA with no specificity for defined HNA antigens is a serological hallmark of autoantibodies observed in patients with autoimmune neutropenia.^16^ Based on this observation, these antibodies will be referred as “neutrophil autoantibodies” in this paper. Sera from patients with autoimmune neutropenia were selected in accordance to the GIFT and MAIGA pattern of TRALI sera, as applicable.

### 3.2 Neutrophil autoantibodies can induce ROS production in neutrophils, but not in endothelial cells in vitro, in selected TRALI cases

**FIGURE 1:**
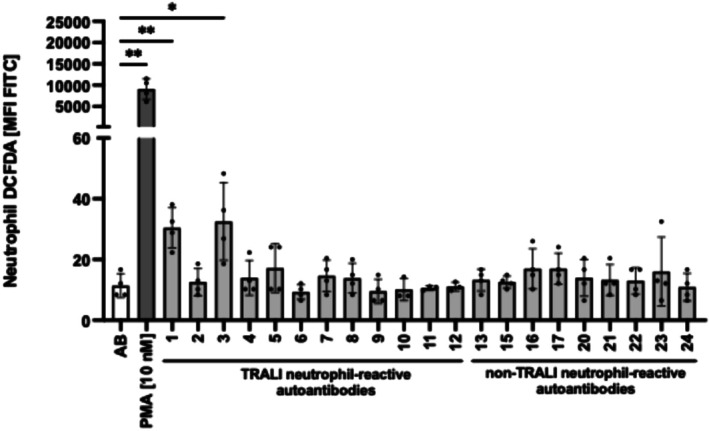
Neutrophils autoantibodies involved in transfusion‐related acute lung injury (TRALI) cases induce reactive oxygen species (ROS) production in neutrophils. ROS inducing effects of human IgG binding (from two groups; TRALI serum with neutrophils autoantibodies and non‐TRALI neutrophils autoantibodies) were compared in 2',7'‐Dichlorodihydrofluorescein diacetate (DCFDA) assay. Isolated neutrophils (1 × 10^6^) were incubated with patient's sera in presence of CM‐H_2_ DCFDA and incubated for 1 h. ROS production was determined by flow cytometry. Phorbol 12‐myristate 13‐acetate (PMA) and pooled AB serum (Negative Control serum from a non‐immunized donor with blood group AB) served as positive and negative controls, respectively. Data are presented as histogram column. Data are shown as means ± SEM and the results of four distinct donors. *p < 0.05 and **p < 0.01.

**FIGURE 2:**
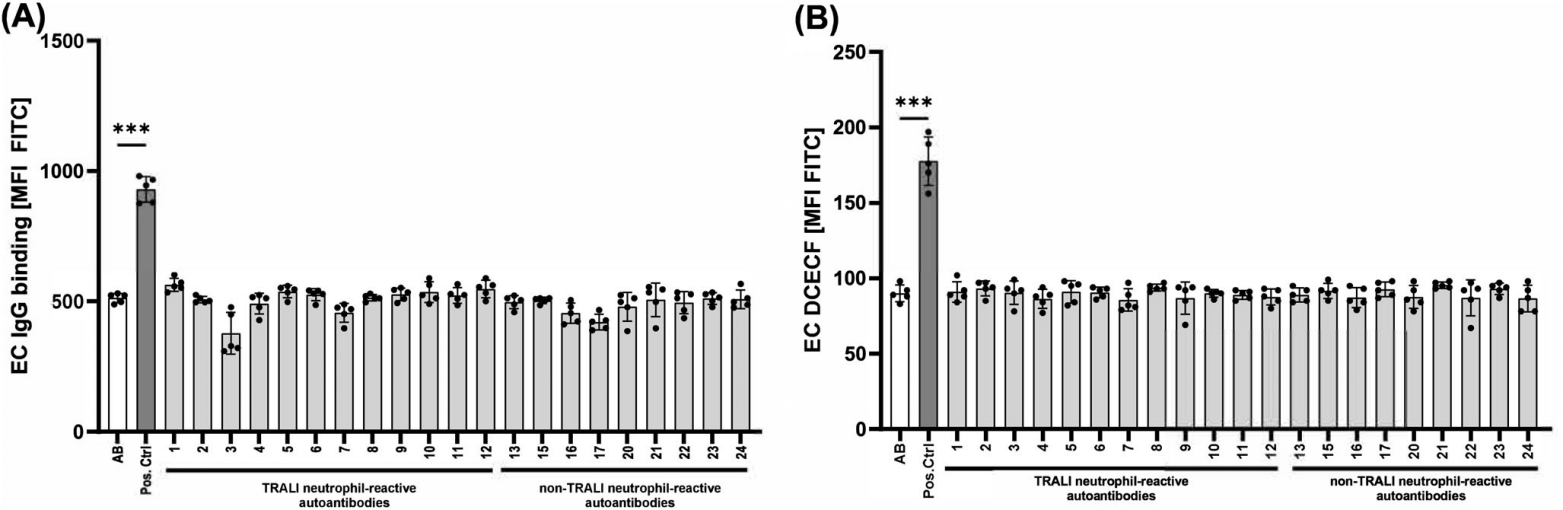
Transfusion‐related acute lung injury (TRALI) sera containing neutrophil autoantibodies fail to bind endothelial cells and induce ROS. Endothelial cells (EC, 3 × 10^5^) were incubated with autoantibodies detected in sera involved in TRALI (1–12) or in patients with autoimmune neutropenia (13–24). Bound human IgG was detected using fluorescein isothiocyanate (FITC)‐labeled anti‐human Fc‐specific antibodies in a flow cytometer (A). To evaluate reactive oxygen species (ROS)‐inducing properties of each serum, endothelial cells were incubated with autoantibodies in the presence of 2′,7′‐dichlorodihydrofluorescein‐diacetate (H_2_DCFDA) and incubated for 1 h. ROS production was determined by flow cytometry (B). Control serum and sera containing alloantibodies reactive with endothelial cells were used as negative and positive controls, respectively. Data are shown as means ± SEM for four distinct cell donors.

Binding of antibodies to neutrophils may lead to cellular activation and ROS production.^17^, ^18^ After incubation of isolated neutrophils (in resting condition or after stimulation with fMLP) with 12 different TRALI sera containing neutrophil autoantibodies in the presence of DCFDA, we evaluated the green fluorescent signal from oxidized DCFDA by flow cytometry. In total, 2/12 sera (16.7%) were capable of inducing ROS (Figure 1, TRALI serum #1 and TRALI serum #3). In contrast, none of the nine autoantibodies obtained from confirmed cases of autoimmune neutropenia were able to induce ROS (Figure 1). Pre‐stimulation with fMLP did not enhance ROS production in neutrophils (data not shown). Our results demonstrate that a minority (*n* = 2) of neutrophil‐reactive autoantibodies from clinical TRALI cases are able to induce neutrophil ROS production. In vivo, released ROS from neutrophils are able to convert resting endothelial cells toward a pro‐inflammatory phenotype and to increase endothelial permeability.^19^, ^20^ However, other studies have documented a role of excessive endothelial ROS production as an alternative inducer of endothelial barrier dysfunction.^21^, ^22^ We therefore also investigated the potential interaction between IgG present in donor sera and endothelial cells. Ea.hy 926 cells did not bind human IgG after incubation with sera from TRALI cases (Figure 2A). In addition, ROS production was not observed after incubation of endothelial cells with TRALI sera or sera containing neutrophil autoantibodies (Figure 2B).

In our previous publications,^21^, ^23^ we compared both Ea.hy 926 and HLMVECs in different assays. To prevent work slowdown due to the slow growth and proliferation rate of HLMVECs, we decided to use only Ea.hy 926 in the current study.

### 3.3 Neutrophil ROS inducing TRALI sera can mediate endothelial barrier permeability in the presence of neutrophils

**FIGURE 3:**
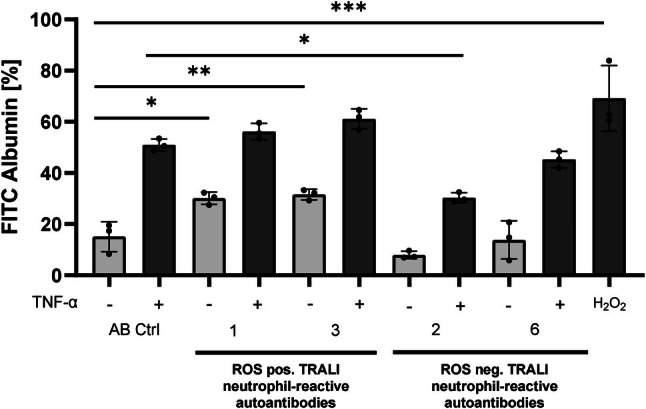
Some autoantibodies detected in transfusion‐related acute lung injury (TRALI) mediate changes of endothelial barrier permeability in the presence of neutrophils. human umbilical vein endothelial cells (HUVECs) (5 × 10^4^) were cultured on fibronectin‐coated polycarbonate membranes in transmigration chambers. In some wells, endothelial cells were stimulated with TNF‐α (20 ng/mL) for 18 h. After 24 h, medium (200 μL) containing albumin‐fluorescein isothiocyanate (FITC), human antibodies (reactive oxygen species [ROS]‐inducing [*n* = 2] and non‐ROS inducing [*n* = 1]) with or without neutrophils (2 × 10^5^) were added to the upper chamber. Every 15 min, fluorescence intensity in the lower chamber was measured by a fluorescent microtiter plate reader (*n* = 4). H_2_O_2_ and pooled control serum served as positive and negative controls, respectively. Data are shown as means ± SEM for four separate donors. *p < 0.05, **p < 0.01, and ***p < 0.0001.

To further evaluate the effects of neutrophil ROS inducing TRALI sera on endothelial barrier permeability, neutrophil ROS inducing positive (serum #1 and serum #3) and neutrophil ROS inducing negative sera (serum #6) were evaluated in a transwell chamber system in the presence and absence of neutrophils. In some experiments, endothelial cells were prestimulated with TNF‐α. The readout in this system was albumin‐FITC flux through an endothelial monolayer. Our results show that only neutrophil ROS‐positive sera (serum #1 and #3) but not neutrophil ROS‐negative sera (serum #6) can induce endothelial barrier break‐down in the presence, but not in the absence, of neutrophils. Although TNF‐α stimulation increased endothelial permeability, this change was not significant compared to the control serum (Figure 3).

### 3.4 Complement fixation

As neutrophils and ROS have been shown to be essential for antibody‐dependent TRALI,^24^, ^25^ in combination with the critical importance of antibody‐dependent complement activation in TRALI,^26^ we investigated a potential contribution of complement fixation for neutrophil‐reactive autoantibodies from TRALI cases.

**FIGURE 4:**
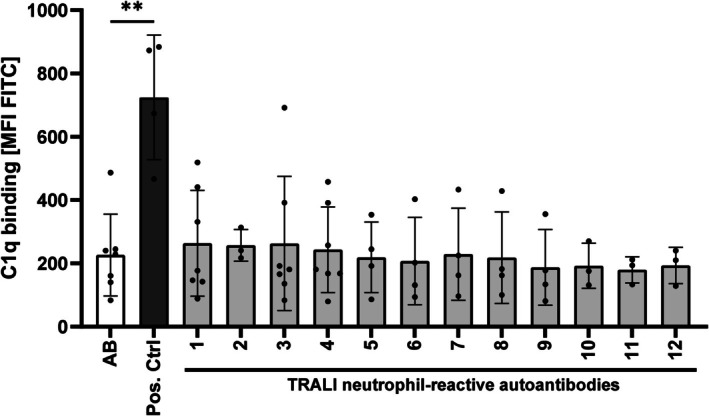
Binding of neutrophils autoantibodies to target antigens expressed on neutrophil fails to fix complement. Complement fixing capacities of human IgG from transfusion‐related acute lung injury (TRALI) sera with neutrophil autoantibodies were evaluated in flow cytometry. Isolated neutrophils (2 × 10^5^) were incubated with human sera in presence of human C1q protein for 30 min. After washing, bound C1q protein was detected by fluorescein isothiocyanate (FITC)‐labeled anti‐human C1q antibody in flowcytometry. Control serum and serum containing complement fixing alloantibodies served as negative and positive controls, respectively Data are shown as means ± SEM for four separate donors. **p < 0.01.

To achieve this, isolated neutrophils (in resting condition or after stimulation with fMLP) were incubated with human sera containing either autoantibodies or alloantibodies against neutrophils (positive control), followed by incubation with C1q. No C1q was fixed on neutrophils that were incubated with autoantibodies or the negative control (heat‐inactivated control serum). However, sera containing alloantibodies (as positive control) could fix C1q (Figure 4). Additionally, pre‐stimulating neutrophils with fMLP did not improve the effectiveness of antibodies to induce ROS or to fix the complement on neutrophil surface (data not shown).

## 4 DISCUSSION

TRALI remains an important differential diagnosis following the occurrence of transfusion‐associated acute respiratory distress. Risk mitigation strategies have not only led to a decrease in the overall frequency of TRALI but, according to preliminary data, also to a decrease in the proportion of “classical” cases of antibody‐dependent TRALI.^27^ Investigating cases of suspected TRALI, in our hands most of the serum from donors does not contain HNA alloantibodies. In many cases, only anti‐HLA antibodies are present. Rarely, we do find antibodies against specific HNAs. However, we sometimes observe a reaction pattern that is reminiscent of autoantibodies against neutrophils such as in autoimmune neutropenia. Here, we demonstrate that (i) autoantibodies against neutrophils can occasionally be found in donor blood involved in TRALI induction; (ii) these neutrophil‐reactive autoantibodies bind to neutrophils, but not to endothelial cells; (iii) and a fraction of these autoantibodies can activate neutrophils and induce ROS; and (iv) the neutrophil ROS inducing autoantibodies are able to induce endothelial barrier permeability in a simplified in vitro model of TRALI, without complement activation.

In this manuscript, antibodies in TRALI sera defined by serological characteristics are termed “autoantibodies” which is based on broad neutrophil binding without specific reactivity to a defined HNAs. They may differ from autoantibodies observed in patients with low neutrophil counts. Autoantibodies against neutrophils likely bind all neutrophils regardless of their antigen type, although there appears to be some modification in binding based on FcγRIIa and FcγRIIIb receptor polymorphisms which alters autoantibodies' affinity to target antigen and affects therefore the phagocytosis efficiency.^15^, ^28^ “Typical” autoantibodies are observed in children between the age of 6 months and 4 years of life diagnosed with autoimmune neutropenia, a disorder that usually goes into spontaneous remission. Although antibodies may gradually decline in titer over time,^16^ no clear correlation between antibody levels, extent of neutropenia, and frequency or severity of infectious complications has been demonstrated. Over the years, several groups have reported autoantibody‐dependent effects on neutrophils that go beyond simple cell clearance including impairment of phagocytotic activity,^29^ complement activation,^16^ neutrophil apoptosis,^30^ interference with neutrophil motility,^31^ and interference with ROS production, mostly reported as a depressed release of ROS.^30^, ^32^, ^33^

The detection of autoantibodies in sera from donors whose blood components triggered TRALI raises the question of how such autoantibodies may lead to acute lung injury. Our investigation shows that few (2/12) autoantibodies are capable of activating neutrophils, an observation that we already know from alloantibodies against neutrophils,^17^ but which has to the best of our knowledge not been described for autoantibodies in autoimmune neutropenia. In fact, 9/9 autoantibodies from patients with autoimmune neutropenia were unable to activate neutrophils, including the two sera that showed a similar reaction pattern to the activating TRALI sera. While complement activation was not systematically studied before 2019,^34^ it has become clear that complement is critically required and is important in classical TRALI.^26^, ^35^ In the current study, however, we did not observe a role for complement suggesting that neutrophil‐reactive autoantibody mediated TRALI may have different pathophysiological features in TRALI, although activation of neutrophil ROS was identified. Our in vitro analysis showed that no “TRALI” autoantibodies could fix complement. Considering the limitations of mono‐cellular in vitro assays such as ours, we cannot exclude the possibility that complement plays a role in the mechanism of neutrophil activation by autoantibodies.

TRALI induction in recipients follows a two‐hit model,^1^, ^3^ whereby patient predisposition (the first hit) and transfusion (the second hit) combine to reach a threshold capable of inducing TRALI.^6^ Studies have accordingly detected increased levels of TNF‐α in TRALI patients compared to the control group.^36^ The current study aimed to replicate the ‘first hit’ by pre‐stimulating endothelial cells and granulocytes with TNF‐α and fMLP, respectively. However, fMLP pre‐stimulation did not affect complement fixation or neutrophil ROS production. In the permeability assay, pre‐stimulation with TNF‐α increased endothelial permeability. Nevertheless, no significant differences in permeability were observed between autoantibody‐stimulated neutrophils and controls.

Others have demonstrated the presence of “autoimmune‐like” antibodies in systematic screening studies in approximately 0.6% of male blood donors.^37^ If 20% were neutrophil‐activating, roughly 1:1.000 male donors would be capable to induce TRALI.

It should be noted that the mere detection of an antibody from donor material, even if this antibody has some in vitro activity, does not mean that it is causative for TRALI. For HLA antibodies, for example, it has been shown that only a minority of recipients with matching HLA antigens actually develop TRALI.^38^ The occurrence of TRALI can follow a threshold model in which the sum of the recipient's baseline health status and the antibodies' concentration and capability precipitates TRALI when the threshold is overcome.^6^ Unfortunately, we do not have the clinical details from the TRALI patients that were investigated in this study.

In conclusion, we demonstrate here that autoantibodies against neutrophils are able to contribute to TRALI in selected, but not all, investigated TRALI cases. The lack of other effector cells in our in vitro analysis affects the validity of this new observation. Further analysis of the effects of donor neutrophil autoantibodies on TRALI induction will require a multicellular approach, increased sample size of TRALI cases, and additional studies in animal models of TRALI.

## CONFLICT OF INTEREST STATEMENT

The authors declare no conflicts of interest.

## Data Availability

The data that support the findings of this study are available on request from the corresponding author. The data are not publicly available due to privacy or ethical restrictions.
